# The Effect of Anti-rheumatic Drugs on the Skeleton

**DOI:** 10.1007/s00223-022-01001-y

**Published:** 2022-06-30

**Authors:** B. Hauser, H. Raterman, S. H. Ralston, W. F. Lems

**Affiliations:** 1 Rheumatic Disease Unit, Western General Hospital, Edinburgh, UK; 2 Rheumatology and Bone Disease Unit, Centre for Genomic and Experimental Medicine, Institute of Genetics and Cancer, University of Edinburgh, Edinburgh, UK; 3 Department of Rheumatology, Northwest Clinics, Alkmaar, The Netherlands; 4 Amsterdam Rheumatology and Immunology Centre, Amsterdam, The Netherlands

**Keywords:** Anti-rheumatic treatment, Rheumatoid arthritis, Bone mineral density, Fracture risk, Erosion repair

## Abstract

The therapeutic armamentarium for rheumatoid arthritis has increased substantially over the last 20 years. Historically antirheumatic treatment was started late in the disease course and frequently included prolonged high-dose glucocorticoid treatment which was associated with accelerated generalised bone loss and increased vertebral and non-vertebral fracture risk. Newer biologic and targeted synthetic treatments and a combination of conventional synthetic DMARDs prevent accelerated systemic bone loss and may even allow repair of cortical bone erosions. Emerging data also gives new insight on the impact of long-term conventional synthetic DMARDs on bone health and fracture risk and highlights the need for ongoing studies for better understanding of “established therapeutics”. An interesting new antirheumatic treatment effect is the potential of erosion repair with the use of biologic DMARDs and janus kinase inhibitors. Although several newer anti-rheumatic drugs seem to have favorable effects on bone mineral density in RA patients, these effects are modest and do not seem to influence the fracture risk thus far. We summarize recent developments and findings of the impact of anti-rheumatic treatments on localized and systemic bone integrity and health.

## Introduction

**Fig. 1:**
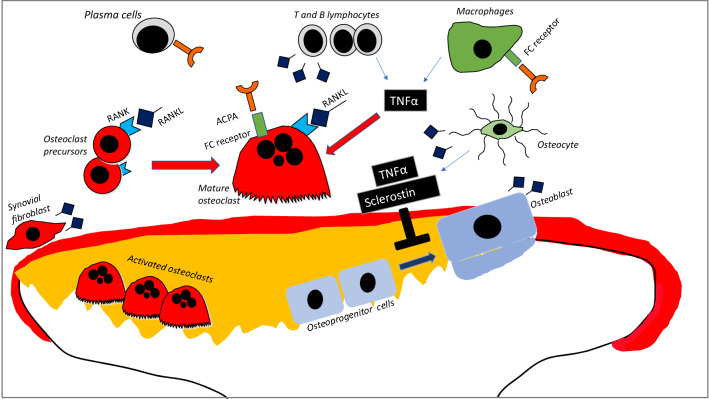
Chronic inflammation and the development of erosions. In the presence of synovitis several cells including T- and B-lymphocytes, synovial fibroblasts, and osteoblasts express RANKL (Receptor activator of nuclear factor kappa-Β ligand). RANKL binds to RANK (Receptor activator of nuclear factor kappa-Β) and promotes osteoclast differentiation and activity. TNFα and other proinflammatory cytokines stimulate osteoclast activity directly and indirectly through stimulation of T cells and increased RANKL production. TNFα reduces bone formation by (**a**) inducing Dickkopf 1 (Dkk-1), which blocks the differentiation of osteoprogenitor cells into osteoblasts and (**b**) inducing the expression of sclerostin in osteocytes, which is a potent downregulator of osteoblast differentiation and activity Anti-citrullinated protein antibodies (ACPA) bind to inflammatory cells (macrophages) and propagate inflammation and can stimulate osteoclasts directly through FC receptors

Rheumatoid arthritis is a chronic inflammatory condition characterized by inflammation of the synovial tissue which can lead to bone and cartilage destruction. Bone erosion, joint damage and destruction play a significant contributory role to functional disability associated with longstanding RA [1]. Both men and women with RA are at significantly higher risk of developing osteoporosis compared to healthy controls [2–4]. Consequently, the fracture risk in patients with RA is at least double of that in the general population [5, 6]. The factors contributing to accelerated systemic bone loss in RA are chronic inflammation, relative immobility, antibody positivity, and glucocorticoids [2, 7]. Chronic inflammation breaks the balance of bone formation by osteoblasts and bone resorption by osteoclasts. The production of pro-inflammatory cytokines such as TNFα and IL-6 in RA stimulate osteoclastogenesis and osteoclast activity directly and indirectly through stimulating receptor activator of nuclear factor kappa-B-ligand (RANKL) expression. Pro-inflammatory cytokines suppress bone formation through stimulating the production of Dickkopf-related protein (DKK1) and Sclerostin (SCL) which suppress osteoblast activity [7, 8].The pathogenesis of localized bone loss and the formation of erosions is depicted below in Fig. 1.

Recent treatment advances and therapeutic strategies focus on rapid control of inflammation, to prevent erosions and irreversible tissue damage and to minimize therapeutic side effects [9]. Over the last 10 years, we have also gained insight into the role of autoantibodies such as anti-citrullinated protein antibodies (ACPA) and osteoprotegerin antibodies on bone turnover [10] [11, 12], phenomena which may be influenced by therapeutic B or T cell inhibition. As we have gained better understanding of the crosstalk between immune system and bone homeostasis, the so-called”osteo-immunology”[13] we expect that the progress in inflammatory disease therapeutics translates to improved bone health and in particular to a reduced burden of osteoporosis as co-morbidity of rheumatoid arthritis.

We will summarize new data on the prevention and repair of bone erosion and will evaluate whether the therapeutic progress is reflected on systemic bone loss and fracture risk in rheumatoid arthritis.

## Conventional Synthetic DMARDS

In the second half of the twentieth century, RA treatment was based on glucocorticoids (GC) and conventional synthetic DMARDs (csDMARDs) including hydroxychloroquine, sulfasalazine, methotrexate, azathioprine, and sodium aurothiomalate (gold salts). Whereas gold salts virtually disappeared as treatment option for RA, other csDMARDs are still used commonly in RA treatment regimes. CsDMARDs in particular methotrexate is first line therapy for the treatment of RA as recommended in EULAR guidelines [14]. In view of efficacy, safety profile and their cost effectiveness csDMARDs will continue to be used in the foreseeable future either as mono-therapy or combination treatment [14]. Although many modern RA treatment strategies aim for GC free remission, GC remain important therapeutics in rheumatoid arthritis, particularly when given short-term and early in the course of the disease. The effects of low-dose GC may be much less deleterious than the effects of chronic high dose GC treatment and indeed a recently published randomized controlled trial on Glucocorticoid LOw-dose in RheumatoId Arthritis (GLORIA) [15] demonstrated no statistical difference in symptomatic fracture rates between patients with established RA treated with 5 mg of Prednisolone or placebo. Further bone health outcomes demonstrated a small but significant lumbar spine BMD loss (-1%) in the Prednisolone group but no differences in hip BMD change between the Prednisolone and placebo group after a mean treatment time of 19 months. The study, however, did show significant better disease activity and less joint damage progression in the Prednisolone group compared to the placebo group suggesting a favorable benefit harm balance of low-dose Prednisolone treatment in elderly RA patients. It is beyond the scope of this review to summarize further the impact of GC on bone.

### Methotrexate: Effective in Preventing Bone Erosions, Unknown Effects on Bone Turnover

Low-dose Methotrexate is the cornerstone therapy in Rheumatoid Arthritis [14]. Its mechanism of action as low-dose therapy is not fully understood but actions such as the inhibition of purine and pyrimidine synthesis, translocation of nuclear factor-κB (NF-κB) to the nucleus, and signaling via the Janus kinase (JAK)–signal transducer and activator of transcription (STAT) pathway are thought to contribute to its anti-inflammatory properties [16]. Methotrexate in combination with GC has been shown in the COBRA-light trial and several other trials to prevent bone erosion and joint destruction in the long term [17–19].

The impact of Methotrexate on systemic bone loss and bone turnover, however, is less well understood. A case–control observational study compared the bone mineral density (BMD) of 60 patients with RA and Psoriatic Arthritis (PsA) who took low-dose MTX for 6 years to that of control patients of similar age, gender, and disease activity who were not treated with Methotrexate. The study did not reveal a significant difference between the groups [20]. Further 46 premenopausal RA patients were started on MTX or SSZ and BMD was measured after 12 and 18 months, again no difference in BMD was detected [21]. More recent data derive from the Women Health Initiative (WHI) observational study which followed 1201 women with RA who were or were not started on csDMARDs up to approximately 6 years. There was no significant difference in self-reported clinical fracture rate between patients who have or have not been started on SSZ or on MTX.

**Fig. 2:**
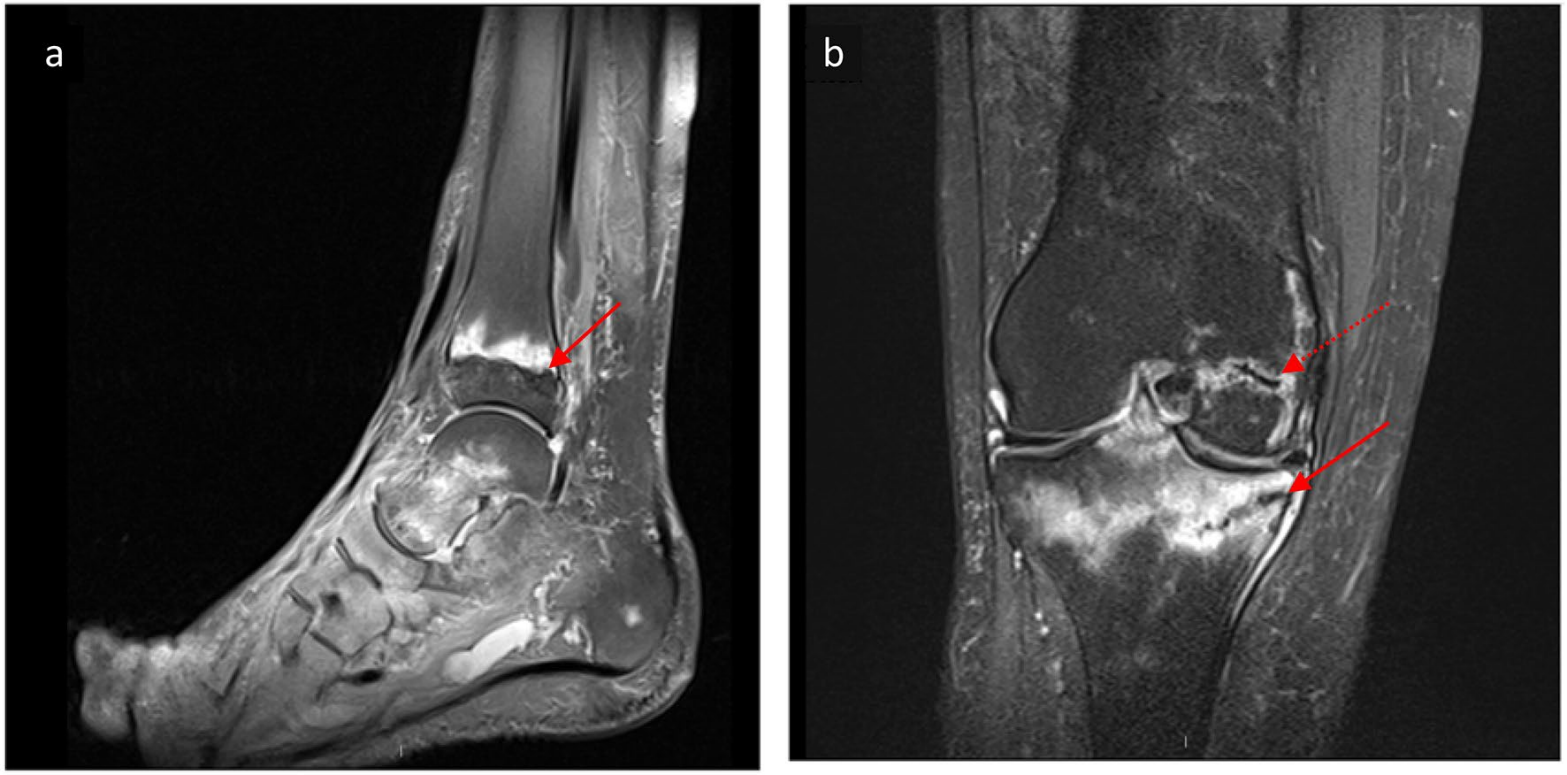
Radiographic features of Methotrexate osteopathy. MRI images of rheumatoid arthritis patients on long-term methotrexate patients who developed insufficiency fractures with band-like fracture lines along the growth plate **a** red arrow points to distal tibia metaphysis insufficiency fracture **b** upper (dotted) red arrow points to distal femur metaphysis fracture and lower (continuous line) red arrow points to proximal tibia metaphysis fracture (Color figure online)

Over the past 2 years an increasing number of patients on long-term MTX were described who developed multiple, frequently bilateral, insufficiency fractures of calcaneus and/or the metaphysis of distal and proximal tibia and distal femur, so called “MTX osteopathy” [22, 23]. Typical MRI findings of methotrexate associated insufficiency fractures are shown in Fig. 2. Methotrexate osteopathy was first described in 1970 when five cases of children developed distal femoral and tibial insufficiency fractures whilst receiving prolonged MTX therapy for acute lymphatic leukaemia [24]. To date about 80 cases have been described with this condition [22, 23, 25]. One of the potential mechanism was thought to be MTX-induced suppression of osteoblast activity [26]; however, this effect was only shown in animal experiments (rats) and to our knowledge low-dose MTX-induced osteoblast suppression has not been replicated in humans. The fact that MTX does not seem to influence BMD [20, 21] points towards a possible idiosyncratic drug reaction of bone cells or bone turnover to MTX. A recently published systematic review on Methotrexate osteopathy summarized that amongst the reported cases about two-thirds of patients discontinued Methotrexate after the diagnosis of Methotrexate osteopathy; however, subsequent fracture healing was rarely reported. Out of the 15 cases in whom fracture healing was reported at least 53.3% had stopped MTX [25].

The previously mentioned WHI study, however, did not detect any difference in lower limb fractures, in MTX users vs non-users [27]. This may be due to the fact that insufficiency fractures are frequently under- or misdiagnosed in patients with rheumatoid arthritis [28]. Insufficiency fractures of the foot and ankle joint in patients with rheumatic diseases are a relatively common finding and a cross-sectional study of a tertiary Rheumatology centre found that out of 1471 foot and 281 ankle MRIs in patients with rheumatic diseases, 7.4% reported on insufficiency fractures. Methotrexate use, low BMI, and low BMD were associated with insufficiency fractures but only half of the patients who were diagnosed with an insufficiency fracture were taking Methotrexate at the time [29]. Further studies are required to investigate the mechanism of insufficiency fractures in patients with rheumatic diseases.

### Sulfasalazine-Likely Little Effect on Bone Health

A cross-sectional study of 104 male RA patients has found a positive association between Sulfasalazine (SSZ) use and trochanteric BMD [30]. A more mechanistic insight gave Jin et al. [31] who demonstrated that SSZ inhibits SCL7A11-enhanced differentiation of mesenchymal stem cells by modulating BMP2/4 expression. The group further showed that in a murine model SSZ treatment in ovariectomized mice attenuated bone loss. Of interest is also a prospective cohort study of pregnant women with RA and follow-up of the children of these mothers. BMD measurements of the children between 5 and 10 years showed that children whose mothers were treated with SSZ during pregnancy had a higher total body BMD [32] compared to children of mothers who were not treated with SSZ. Subsequent studies ,however, do not confirm any beneficial impact of SSZ on fracture risk [27].

### Hydroxychloroquine-Conflicting Results

Hydroxychloroquine (HCQ) as RA treatment is frequently used as combination treatment with MTX or SSZ. Both et al. observed higher BMD in Sjogren patients compared to healthy controls [33] which was hypothesized to be a result of HCQ treatment; another explanation is that the positive effect on BMD is caused by low disease activity due to the anti-inflammatory effects of HCQ. Subsequent in vitro studies demonstrated no difference in osteoclast formation between HCQ treatment and control medium; however, HCQ may have an impact on osteoclast activity as HCQ treated osteoclasts resorbed significantly less bone than control osteoclasts [34]. When tested on osteoblasts HCQ treatment was found to decrease human mesenchymal stem cells derived osteoblast differentiation and mineralization in vitro [35]. The WHI observational study in RA patients taking HCQ have not found a difference in fracture rate in comparison to HCQ non-users [27].

There is no or minimal data on BMD or fracture risk for leflunomide or azathioprine available.

## Biologic DMARDS

### TNF Inhibitors-Great Expectations

At the start of the twenty-first century, RA treatment changed dramatically. The introduction of biologic DMARDS (bDMARDs) in particular TNF inhibitors (TNFi) and the change in RA treatment strategy, which included early aggressive treatment, treat to target strategy and to aim for disease remission, improved long-term RA outcomes substantially [36].

TNFα is a key inflammatory cytokine propagating chronic inflammation. TNFα stimulates osteoclastogenesis and osteoclast activity directly [37] and through stimulation of RANKL expression [38]. TNFα also suppresses osteoblast formation through suppression of RUNX2 and osteocalcin [39]. TNF inhibitors (TNFi) have been proven to be efficient in preventing bone erosion, joint destruction with resulting disabilities, and to improve general health with reduced cardiovascular risk [40–43]. Comprehensive reviews on the effect of TNFi on BMD were published before [44–47]. A summary of the overall effects of bDMARDs and targeted synthetic (ts) DMARDs on BMD and erosion repair is shown in Table 1.

**Table 1:** Summary table of the effect of bDMARD and tsDMARDs on spine, hip and hand BMD and on erosion repair

	Spine BMD	Hip BMD		Hand BMD		Erosion repair*	
			Ref		Ref		Ref
TNFi	+/--	+/-	[51] [57] [58] [47]	+/--	[51] [48, 59]	+	[52]
Rituximab	+/-	+/--	[60, 61]	×		x	
Abatacept	+/-	++/-	[57] [62]	×		+	[63]
IL6i	+/-	++/-	[64–66]	×		++	[67, 68]
JAKi	+/-	+/-	[69]	×		?+	[70]

*TNFi tumour necrosis factor inhibitors, IL6i* *interleukin 6 inhibitors, JAKi* *janus kinase inhibitors; BMD* *bone mineral density;*

*Erosion repair-assessed by HR-pQCT or MRI, *× no data available,*±*stable*, ±—*more studies demonstrating BMD loss than gain*,+  ± *most studies demonstrating BMD stabilisation or gain*, +*evidence of erosion repair*, +  + *evidence of erosion repair which is superior to other bDMARDs*, *?*  +*preliminary data from a small sample size only*, *Further detailed table on the impact of bDMARDs on bone mass and bone turnover markers is published by Zerbini *et al. [47]

Initial observational studies demonstrated that the use of regular intravenous TNFi infusions (infliximab) reduces the usually occurring rapid bone loss in RA and the favorable effect on BMD in RA patients was later replicated for the subcutaneous TNFi, adalimumab [48, 49]. The anti-inflammatory effect of TNFi was, as expected, associated with a reduction of the bone resorption marker (serum CTX) and an increase of bone formation marker (osteocalcin) [49]. Further results on BMD by TNFi use are modest with stabilization of BMD as shown by initial BMD assessments in the BeST study [50], observations which were later confirmed by a meta-analysis by Siu et al. [51], see Table 1. Recent systematic reviews on the impact of bDMARD use on fracture risk have not demonstrated a difference in the fracture rate in RA patients treated with or without TNFi [52] and between different bDMARDs [53]. A recent cohort study looked at 4265 RA patients who were bDMARD users and propensity score matched to the same number of bDMARD naive patients. During an average follow-up period of 4.4 years, 229 patients on bDMARDs-sustained osteoporotic fractures which was similar to the bDMARD naïve group in which 205 patients after 3.7 years sustained a clinical fracture. The authors concluded that the use of bDMARDs was not associated with a reduced risk of osteoporotic fractures in both women and men [54].

Clearly one can argue that patients requiring bDMARDs have a higher fracture risk than patients without bDMARDs, therefore no difference in fracture rate could be regarded as consequence of bDMARD treatment. In addition also, beneficial effects of TNFi use on fracture risk was shown in a large longitudinal observational registry study of 11,412 patients with RA. The study demonstrated that TNFi and statin use was associated with decreased vertebral fractures whereas opioids and glucocorticoids were associated with increased risk of any fracture in patients with RA [55].

Remarkably, one of the first reports of erosion repair was given by Finzel et al. [56] who described in RA patients reduction of bone erosion depth and sclerosis at the base of the lesions when assessed by consecutive µCT scans after one year of treatment with TNFi, findings which were significantly different from the MTX treatment group.

### Rituximab-Minimal BMD Change

A number of clinical and translational studies have demonstrated pathways of autoantibody driven bone loss [10]. B-cell inhibition reduces the number of circulating B-cells, plasma cells and immunoglobulins [71]. Rituximab is similarly effective as TNF inhibitors in preventing erosions and has been proven to be effective in patients who have inadequately responded to TNFi [72]. Its effect on systemic bone loss has not been studied to great extent. One study, however, monitored BMD changes in 45 pts who received RTX, no significant difference in BMD was found after 12 months of treatment. However, there was a significant increase in procollagen type 1 amino-terminal propeptide (P1NP) and bone specific alkaline phosphatase (BAP) found, which are both biomarkers of bone formation (median change from baseline to 12 months; P1NP 11.3 μg/L, 95% CI −1.1, 24.8 *p* = 0.025; BAP 2.5 μg/L, 95% CI −1.2, 3.6 *p* = 0.002). Analysis of bone resorption markers did not find any significant change after 12 months of Rituximab treatment [60]. A recent small retrospective study has found that after 18 months of RTX treatment the lumbar spine BMD has significantly improved in 20 postmenopausal women with RA (+ 7%, *p* = 0.0029), the femoral neck BMD remained stable [61]. These results suggest that studies with a longer observation period maybe required to detect a significant BMD change.

### Abatacept-Little Data, Favorable Effects on Bone

Abatacept a cytotoxic T-lymphocyte protein-4 Immunoglobulin (CTLA4-Ig) is a chimeric protein that acts as T-cell co-stimulation modulator. Similar to TNFi and Rituximab, Abatacept prevents erosions and structural damage in RA to a great extent [73, 74]. Also, erosion repair was reported in a small number of patients (11%) who received intravenous abatacept who were assessed with MRI scans of hands after 12 months of treatment [63]. A reduction of systemic bone loss with the use of Abatacept was shown in a murine model of hyperparathyroidism [75]. Tada et al. [62] demonstrated in a prospective non-randomized cohort study that patients on Abatacept had a marginal higher BMD gain at the femoral neck when compared to RA patients who had been treated with other bDMARDs. A recently published longitudinal study on RA patients who were followed up for 3 years, compared the effect of cDMARDs, TNFi, and Abatacept treatment on BMD. Patients who received Abatacept had a stable spine and hip BMD over 3 years whereas patients on cDMARDs or TNFi lost BMD over time[57].

### IL6 Inhibition-Erosion Repair

IL6 blockade is an effective strategy to counteract inflammation and the development of bone erosions in patients with RA [76]. IL-6 is over-expressed in the inflamed synovium of RA patients and increased concentrations of IL-6 are found in serum and synovial fluid of these patients. Many inflammatory cells including macrophages, osteoblasts and T cells have the ability to express IL-6 [77, 78]. Interestingly, the addition of IL-6 to murine and human osteoclast cell cultures inhibits osteoclastogenesis [79]; however, in the setting of inflammatory arthritis IL-6 is thought to have a pro-osteoclastogenic effect mediated by increased RANKL production by osteoblasts and by direct stimulation of osteoclast precursors through gp130 signaling [78]. The use of tocilizumab over a 1 year period in a RA cohort was associated with a mild increase of BMD in patients with methotrexate resistant active rheumatoid arthritis with underlying osteopenia [66]. Further analysis of 76 RA patients who received combination treatment of methotrexate and tocilizumab over 48 weeks did not demonstrate any significant BMD improvement but stabilisation. The study, however, noted an overall decrease in DKK1 and an increase in bone formation markers [65]. A more recent prospective non-controlled cohort study demonstrated a modest increase in femoral neck BMD and reduction in bone resorption markers CTX in ACPA positive RA patients treated with 2-year tocilizumab. Interestingly, a positive BMD effect of tocilizumab was not observed in ACPA neg RA pts [64].

Regarding fracture healing, recent murine models demonstrated the importance of IL-6 classic signalling for bone repair and suggest that IL-6 blockade may delay fracture healing [80]. To our knowledge there is no data on the use of IL-6 inhibitors and fracture healing in humans. The use of high resolution peripheral QCT (HR-pQCT) in the assessment of bone erosions has allowed a closer insight in development of cortical erosions. A study comparing HR-pQCT images of metacarpal heads and radii of 33 RA patients on tocilizumab monotherapy with 33 patients on a combination therapy of adalimumab (TNFi) and MTX has shown better erosion repair in the tocilizumab group than in the comparator group [67].

Erosion repair is mainly shown on HR-CT [67, 68] or MRI scans [63] in research setting, see Table 1. Only a few case reports of mainly systemic juvenile idiopathic arthritis patients treated with tocilizumab demonstrate erosion repair on conventional radiographs [81–84] however no large-scale research trial has demonstrated significant erosion repair with IL-6 inhibition on X-rays. Although the clinical significance of erosion repair seen on HR-pQCT is unclear, these findings may help to stratify treatment in patients at high risk of erosive progression.

### JAK Inhibitors-Bone Repair with Small Molecules

Janus kinase (JAK)-mediated cytokine signaling is an important target for the treatment of inflammatory diseases including rheumatoid arthritis (RA). JAK inhibition reduces disease activity in RA as effectively as bDMARDs and in patient’s refractory to bDMARDs, targeted synthetic DMARDs (tsDMARDs) such as JAK inhibitors were shown to be more effective in reducing disease activity than TNFi or abatacept [85–87]. Until recently, the role of the JAK/signal transducers and activators of transcription (STAT) pathway in bone turnover was fairly unknown.

Adam et al. [70] showed that in vitro JAK inhibition boosts osteoblast function but does not appear to make a difference to osteoclast proliferation or function. Furthermore, in mouse models of osteoporosis and inflammatory arthritis the use of two different JAK inhibitors (Tofacitinib and Baricitinib) mitigates the bone loss induced by ovariectomy or chronic inflammation. Although above paper has also included the example of two patients who received Tofacitinib and HR-pQCT images of the metacarpophalangeal joints suggests erosion repair, further larger studies are necessary to evaluate (a) the extent of erosion repair and (b) if erosion repair detected on HR-pQCT allows the repair of affected joints with JAK inhibition. Additionally, a recent small prospective study with 24 patients who were started on Tofacitinib demonstrated stable areal and volumetric BMD after 1 year of treatment with Tofacitinib with a significant decrease in bone resorption markers (CTX) after 6 months of treatment [69].

Clearly early data on JAK inhibition signals promising data in regard to erosion repair and preventing systemic bone loss. However, recent reports on post-authorization safety trial outcomes on Tofacitinib which demonstrated increased risk of major cardiovascular events and non-melanoma skin cancer in the Tofacitinib group when compared with TNFi highlights the need for careful assessment of risk and benefits of treatments offered to the patients [88].

### IL-17 Inhibitors-Inhibition of Structural Damage

The cytokine IL-17 is a product of mast cells and Th17 cells, which are found in abundance in the inflamed synovium. IL-17 potently induces RANKL expression in synovial fibroblasts and osteoblasts and stimulates innate immune cells to express IL-1 and TNF [89]. Although IL-17 is a potentially attractive therapeutic target in RA, randomised phase 2 placebo controlled trials of IL-17 inhibitors in RA have yielded disappointing results such that these agents are not currently used in the treatment of RA [90, 91]. The IL-17/IL-23 axis is ,however, an important and established treatment target in spondyloarthropathies. Both IL-17 and IL-23 inhibitor reduce radiographic progression of peripheral joints in patients with psoriatic arthritis [92]. Furthermore IL-17 inhibition (IL17i) may reduce new bone formation in axial spondyloarthritis as 2 year treatment with IL17i (secukinumab) showed that the vast majority (97.1%) of the treated patients remained syndesmophyte-free [93] :however, head to-head studies comparing different biologic therapies in prevention of new bone formation in axial spondyloarthritis are outstanding. In regard to the systemic bone effect, a post hoc analysis of secukinumab treatment in ankylosing spondylitis showed an increase of lumbar spine BMD by 2.6% after 2 years of treatment with no radiographic progression but no relevant effects on bone turnover markers [94]. Similar to other biologic treatments, the impact of IL17i on BMD and fracture risk is at best modest and will not replace antiresorptive osteoporosis treatment if indicated.

## Osteoporosis Medication and Erosion Repair

Denosumab is a monoclonal antibody that binds and inhibits RANKL resulting in reduced osteoclast formation and activity. Denosumab was developed as osteoporosis treatment and the pivotal trial in 2009 demonstrated fracture risk reduction at all sites over 36 months [95]. Subsequent studies demonstrated that the addition of denosumab to methotrexate in patients with early rheumatoid arthritis inhibited the progression of joint erosions when compared to placebo and methotrexate [96]. A post hoc analysis of pQCT scans of RA patients treated with either alendronate or denosumab demonstrated a significant decrease in erosion size in the denosumab group whereas erosions in patients who were treated with Alendronate progressed [97]. A further large randomized controlled study, the DESIRABLE study, investigated the effect of 3 monthly and 6 monthly denosumab on erosion progression in patients with RA who were treated with csDMARDs. The overall erosion score when assessed radiographically with the modified total sharp score progressed in all groups ;however, it was significantly smaller in the denosumab groups when compared to placebo. The study, however, did not report on any notable erosion repair in the denosumab group [98]. A recent systematic review on denosumab use in patients confirmed its beneficial impact on lumbar spine and hip BMD and on prevention of erosions and joint destruction in patients with rheumatoid arthritis [99]. Altogether, denosumab might be an attractive option for RA patients with osteoporosis, since it has been shown to increase BMD, reduce fracture rate, and its positive impact on localized bone loss.

Another osteoporosis medication investigated for its potential action on erosion repair was Teriparatide, intermittent parathyroid hormone treatment, that stimulates bone formation [100]. A randomized clinical trial in established RA patients, however, did not find a beneficial effect of one-year Teriparatide treatment on erosion volume when assessed by computer tomography [101].

## Summary

Patients with RA are at increased risk of developing generalised osteoporosis and to sustain vertebral and non-vertebral fractures during the course of the disease. The main mechanisms for bone loss in rheumatic diseases are thought to be chronic inflammation, relative immobility, and the use of GC. However also in patients, particularly in elderly with early RA a high prevalence (30%) of existing vertebral deformities was observed [102] highlighting the importance for rheumatologists to be vigilant from early on in the disease.

Modern anti-rheumatic treatment is particularly effective in preventing bone erosions and to some extent systemic bone loss. Several observational studies demonstrate that the combination treatment of bDMARDs and csDMARDs over one year can halt or minimize the usually occurring loss in BMD at the spine and hips. The data support that optimal reduction of disease activity not only has direct favorable effects on joint scores and extra-articular signs and symptoms, but also reduces generalised bone loss.

However, BMD gains with biologic anti-rheumatic treatments are at best marginal and so far, do not seem to influence the fracture risk substantially. Additionally, patients with RA may have a background risk of developing osteoporosis independent of the diagnosis of RA. Therefore, it is important to note that anti-rheumatic treatments do not replace osteoporosis treatment in patients with RA. Fracture risk assessment should be an integral part of the regular assessment of all RA patients, even if the rheumatoid arthritis is well controlled or in remission. Patients with osteoporosis or increased fracture risk should be offered anti-osteoporosis treatment in order to reduce fracture risk.

Interestingly, for IL-6 inhibition and, to a lesser extent, for TNFi, abatacept, and JAK inhibition, some data were found that show repair of erosions, an exciting and favorable effect of strong inflammatory suppression on bone health. Erosion repair was only found on detailed imaging such as HR-CT and MRI scans thus far and its clinical significance remains to be established.

## Future Direction

The tremendous success in controlling inflammatory arthritis will likely be beneficial for overall bone health. The focus in treating RA ,however, must remain on the prevention of erosions, structural damage, and systemic bone loss. In clinical practice regular assessment of erosion status, bone mineral density and fracture history will allow the evaluation if current treatment is effective in preventing irreversible joint damage and osteoporosis. The repair of bone erosions seems to be an exciting possible tool in future, and we are interested to await further studies to assess the potential clinical and functional impact of erosion repair in RA patients. Although no anti-rheumatic medication has shown a reduction in fracture risk thus far, recent studies revealing BMD stabilisation and improvements with bDMARD and tsDMARD may translate in a reduction of fracture risk for RA patients in the long run.
